# Analysis of structure indicators influencing 3-h and 6-h compliance with the surviving sepsis campaign guidelines in China: a systematic review

**DOI:** 10.1186/s40001-021-00498-7

**Published:** 2021-03-19

**Authors:** Lu Wang, Xudong Ma, Huaiwu He, Longxiang Su, Yanhong Guo, Guangliang Shan, Xiang Zhou, Dawei Liu, Yun Long, China National Critical Care Quality Control Center Group

**Affiliations:** 1 Department of Critical Care Medicine, Peking Union Medical College and Chinese Academy of Medical Sciences, Peking Union Medical College Hospital, Beijing, 100730 China; 2 State Key Laboratory of Complex Severe and Rare Diseases, Peking Union Medical College Hospital, Chinese Academy of Medical Science and Peking Union Medical College, Beijing, 100000 China; 3 Department of Medical Administration, National Health Commission of the People’s Republic of China, Beijing, 100000 China; 4 Department of Epidemiology and Biostatistics, Institute of Basic Medicine Sciences, Chinese Academy of Medical Sciences (CAMS) &School of Basic Medicine, Peking Union Medical College, Beijing, 100000 China

**Keywords:** Structure indicator, ICU, Compliance, Surviving sepsis campaign guidelines, China

## Abstract

**Background:**

Compliance with the surviving sepsis campaign (SSC) guidelines (C_ssc_) is a key factor affecting the effects of sepsis treatment. We designed this study to investigate the relationships of the structure indicators of ICU on 3 and 6-h *C*_ssc_ in China.

**Methods:**

A total of 1854 hospitals were enrolled in a survey, led by the China National Critical Care Quality Control Center (China-NCCQC) from January 1, 2018, through December 31, 2018. We investigated the 1854 hospitals’ 3 and 6-h *C*_ssc_, including compliance with each specific measure of the 3-h and 6-h SSC bundles. We also investigated the actual level of the structure indicators of ICU, released by China-NCCQC in 2015.The outcomes were in adherence with the SSC guidelines (2016). Monitoring indicators included 3 and 6-h *C*_ssc_.

**Results:**

In the subgroup, the rate of broad-spectrum antibiotic therapy was the highest, and the rate of CVP and ScvO2 measurement was the lowest among the items of 3 and 6-h *C*_ssc_. Structure indicators related to 3 and 6-h *C*_ssc_ include the predicted mortality rate and the standardized mortality ratio (SMR). The relationships between 3 and 6-h *C*_ssc_ and the proportion of ICU in total inpatient bed occupancy, the proportion of acute physiology and chronic health evaluation (APACHE) II score ≥ 15 in all ICU patients were uncertain. There was no relationship of 3 and 6-h *C*_ssc_ with the proportion of ICU patients among total inpatients.

**Conclusions:**

Structure indicators influencing 3 and 6-h *C*_ssc_ in China are the predicted mortality rate and the standardized mortality rate.

**Supplementary Information:**

The online version contains supplementary material available at 10.1186/s40001-021-00498-7.

## Background

Sepsis 3.0 is defined as downregulation of host response after infection and the occurrence of life-threatening organ dysfunction [1, 2]. The overall global burden of sepsis has increased over the past several decades. Recent studies have shown that the mortality from sepsis can be reduced by compliance with the surviving sepsis campaign (SSC) guidelines (*C*_ssc_) [3–5]. However, the *C*_ssc_ in clinical work is only approximately 30–60% [6].

The structure indicators of intensive care unit (ICU) reflect whether the resource allocation of the ICU meets the requirements. At present, there are few studies on relationship between structure indicators and the *C*_ssc_ [7]. We assume that the structure indicators will have an important impact on the *C*_ssc_. Therefore, the aims of this study were the following: 1. to investigate the *C*_ssc_ and structure indicators of ICU in China; and 2. determine the relationships between the *C*_ssc_ and structure indicators of ICU_._

## Methods

### Hospitals

The total number of secondary and tertiary hospitals registered was 7525 across the country in 2018. China National Critical Care Quality Control Center (China-NCCQC) collected detailed data regarding quality control indicators through the database of the National Clinical Improvement System (https://icuqc.console.clinify.cn/dataMonitoring). The data were collected between January 1, 2018 and December 31, 2018. Hospitals with patients of septic shock admitted in ICUs < 20/year and incomplete data were excluded from this study. There were only 6 private specialized hospitals, including 4 tertiary hospitals and 2 secondary hospitals, so they were not included in this survey. At last, 1854 hospitals in China were involved. All of the information from participating hospitals is listed in Table 1 and Additional file 1. The 3 and 6-h *C*_ssc_ are listed in Table 2 and Additional file 2: Figures S1–S4 and Additional file 3: Figures S1–S4.

**Table 1:** Basic information of different types of hospitals

	Hospitals	Beds_hos_	Beds_ICU_	Patients_hos_	Patients_ICU_	Days_hos_	Days_ICU_	Doctors_ICU_	Nurses_ICU_
Public									
General									
Tertiary	877	1,246,054	25,054	49,534,487	1,052,682	449,420,159	7,177,717	13,439	47,193
Secondary	698	389,854	6777	15,164,592	285,984	226,320,512	14,097,614	3991	11,558
Specialized									
Tertiary	153	111,351	4717	5,921,433	206,430	49,887,588	1,654,392	2250	6819
Secondary	21	3369	368	195,704	16,176	1,619,987	75,098	156	379
Private									
General									
Tertiary	41	39,346	725	1,389,714	25,114	15,842,900	157,202	320	1135
Secondary	58	25,768	507	907,210	18,440	7,701,873	118,324	273	815
Total	1848	1,815,742	38,148	73,113,140	1,604,826	750,793,019	23,280,347	20,429	67,899

*Beds*_*hos*_ hospital beds, *Beds*_*ICU*_ ICU beds, *Patients*_*hos*_ Patients admitted in hospitals, *Patients*_*ICU*_ Patients admitted in ICUs, *Days*_*hos*_ Days of hospital bed occupancy by patients, *Days*_*ICU*_ Days of ICU bed occupancy by patients, *Doctors*_*ICU*_ ICU doctor number, *Nurses*_*ICU*_ ICU nurse number

**Table 2:** Compliance of surviving sepsis campaign (SSC) guidelines (Cssc) of different types of hospitals

	3 h *C*_ssc_	3 h *C*_ssc-lac_	3 h *C*_ssc-cul_	3 h *C*_ssc-spe_	3 h *C*_ssc-res_	6 h *C*_ssc_	6 h *C*_ssc-rep_	6 h *C*_ssc-vas_	6 h C_ssc-CVP_
Public									
General									
Tertiary	0.75	0.82	0.75	0.85	0.78	0.64	0.66	0.66	0.49
Secondary	0.75	0.79	0.74	0.83	0.75	0.63	0.65	0.65	0.49
Specialized									
Tertiary	0.70	0.76	0.70	0.80	0.74	0.59	0.67	0.67	0.52
Secondary	0.68	0.86	0.78	0.91	0.79	0.64	0.79	0.70	0.56
Private									
General									
Tertiary	0.76	0.78	0.72	0.87	0.79	0.68	0.61	0.57	0.46
Secondary	0.68	0.82	0.83	0.89	0.84	0.63	0.72	0.68	0.50
Total	0.74	0.80	0.74	0.84	0.77	0.63	0.66	0.66	0.50

*SSC* surviving sepsis campaign, *C*_*ssc*_ compliance of SSC guidelines, *3 h C*_*ssc-lac*_ completion of lactate concentration was determined, *3 h C*_*ssc-cul*_ completion of appropriate routine microbiologic cultures (including blood) be obtained before starting antimicrobial therapy, *3 h C*_*ssc-spe*_ completion of empiric broad-spectrum therapy, *3 h C*_*ssc-res*_ completion of resuscitation with 30 ml/kg crystal liquid, *6 h C*_*ssc-rep*_ completion of repeated measurement of lactate levels in patients with initial hyperlactatemia, *6 h C*_*ssc-vas*_ completion of resuscitation with vasopressor in patients with MAP ≤ 65 mmHg after fluid resuscitation, *6 h C*_*ssc-CVP*_ completion of CVP and ScvO2 were measured in patients with lactate ≥ 4 mmol/L

### Study design

In this study, the structure indicators of ICU were evaluated according to the National Clinical Quality Control Indicators for Critical Care Medicine (2015 Edition) released by the China-NCCQC. Monitoring indicators included the proportion of ICU patients among total inpatients, the proportion of ICU patients out of total inpatient bed occupancy, the proportion of APACHE II scores ≥ 15 in all ICU patients, the predicted mortality rate and the standardized mortality ratio. Each indicator is divided into 4 grades according to the implementation. Each 25% from bad to good is divided into the lowest group, the lower group, the higher group, and the highest group.

The primary end points were the 3 and 6-h *C*_ssc_. Monitoring indicators included 3-h *C*_ssc_ (1. Completion of lactate concentration was determined, 2. Completion of appropriate routine microbiologic cultures [including blood] obtained before starting antimicrobial therapy, 3. Completion of empiric broad-spectrum therapy, 4. Completion of resuscitation with 30 mL/kg crystal liquid) and 6-h *C*_ssc_ [1. Completion of repeated measurement of lactate levels in patients with initial hyperlactatemia, 2. Completion of resuscitation with vasopressor in patients with mean arterial pressure (MAP) ≤ 65 mmHg after fluid resuscitation, 3. Completion of central venous pressure (CVP) and central venous oxygen saturation (ScvO_2_) measured in patients with lactate ≥ 4 mmol/L].

According to the above levels, we investigated the relationships of the structure indicators of ICU on 3 and 6-h *C*_ssc_ in patients with sepsis in China.

The study was conducted in accordance with the *Declaration of Helsinki* (as revised in 2013). The trial protocol was approved by the Central Institutional Review Board at Peking Union Medical College Hospital (NO.: S-K1297) and individual consent for this retrospective analysis was waived. The authors are accountable for all aspects of the work in ensuring that questions related to the accuracy or integrity of any part of the work are appropriately investigated and resolved.

### Data collection

In all of the participating clusters, data were obtained and entered into a web-based data entry system by a local, trained independent research coordinator. Range checks were used to check for inconsistent or out-of-range data, prompting the user to correct or review data entries outside the predefined range. The system also provided predefined logic checks to identify errors or illogical data entries. A data quality meeting was held monthly to review all of the hospital enrollment records and registry data.

### Data analysis

Statistical analysis was performed using SPSS software, version 16.0 (IBM Corp., Armonk, NY, USA). The Kolmogorov–Smirnov test was employed to check whether the data were normally distributed. The results are described as mean ± standard deviation. Comparisons between multiple groups were analyzed by one-way analysis of variance (ANOVA), and pairwise comparisons after ANOVA were conducted using the Tukey multiple comparisons test. All of the statistical tests were two-tailed, and a *P* < 0.05 was considered to be statistically significant.

## Results

### Correlations between the structure indicators of ICU and C_ssc_.

**Fig. 1:**
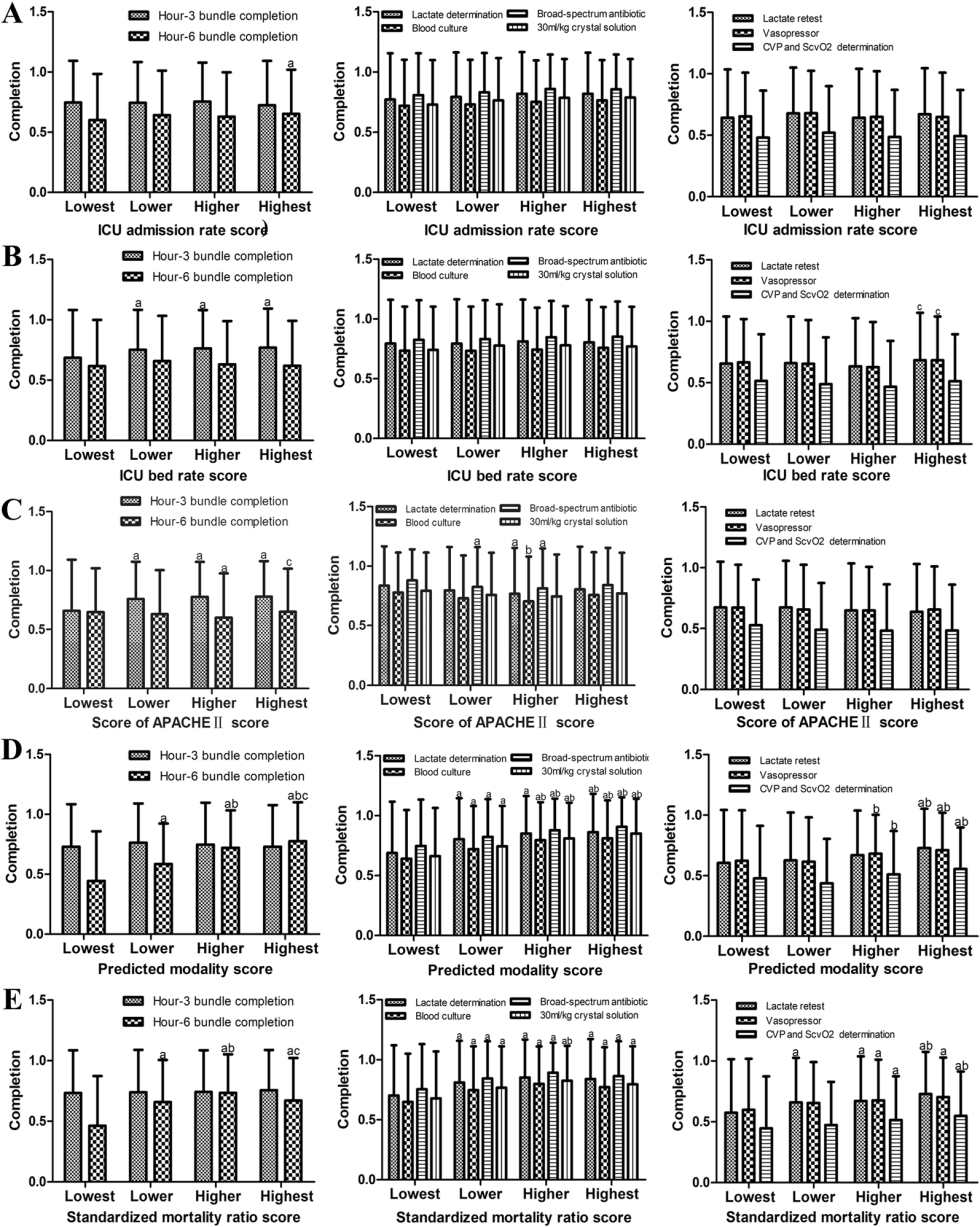
Correlation between the structure indicators and compliance of surviving sepsis campaign (SSC) guidelines (C_ssc_). Each indicator is divided into 4 grades according to the implementation. Each 25% from bad to good is one level, with 0, 1, 2, or 3 points. According to the scores, they are divided into the lowest group, the lower group, the higher group, and the highest group. Results are presented as mean ± SD. a = *P* < 0.05 compared to the lowest group, b = *P* < 0.05 compared to the lower group, c = *P* < 0.05 compared to the higher group. DVT = deep vein thrombosis. ICU = intensive care unit. ICU admission rate = the proportion of ICU patients among total inpatients, ICU bed rate = the proportion of ICU patients out of total inpatient bed occupancy, APACHE II score = the proportion of APACHE II scores ≥ 15 in all ICU patients

No statistically significant difference was found with respect to the proportion of ICU patients among total inpatients. There was no relationship of 3 and 6-h *C*_ssc_ with the proportion of ICU patients among total inpatients (Fig. 1a).

3-h C_ssc_ in the lower, higher and highest group of the proportion of ICU in total inpatient bed occupancy was significantly higher than that in the lowest group (*P* < 0.05) (Fig. 1b). However, same phenomenon was not observed in hour-6 bundle and each sub-index of hour-3 bundle (Fig. 1b). 

In the lower, higher and highest group of the proportion of APACHE II scores ≥ 15 in all ICU patients, 3-h C_ssc_ was significantly higher than that in the lowest group (*P* < 0.05) (Fig. 1c). Completion of empiric broad-spectrum therapy in the lower and higher group was significantly higher than that in the lowest group (*P* < 0.05) (Fig. 1c). However, same phenomenon was not observed in hour-6 bundle_,_ other sub-index of hour-3 bundle and sub-index of hour-6 bundle (Fig. 1c).

6-h C_ssc_ in the lower, higher and highest group of the predicted mortality rate was significantly higher than that in the lowest group (*P* < 0.05) (Fig. 1d). 6-h C_ssc_ in the higher and highest group was significantly higher than that in the lower group (*P* < 0.05) (Fig. 1d). 6-h C_ssc_ in the highest group was significantly higher than that in the higher group (*P* < 0.05) (Fig. 1d). Completion of each sub-index of hour-3 bundle in the lower, higher and highest group was significantly higher than that in the lowest group (*P* < 0.05) (Fig. 1d). Completion of each sub-index of hour-3 bundle in the higher and highest group was significantly higher than that in the lower group except completion of lactate concentration was determined in the higher group (*P* < 0.05) (Fig. 1d). Completion of each sub-index of hour-6 bundle in the higher and highest group was significantly higher than that in the lower group except completion of repeated measurement of lactate levels in patients with initial hyperlactatemia in the higher group (*P* < 0.05) (Fig. 1d). These results indicated that higher predicted mortality rate mean better C_ssc_.

6-h C_ssc_ in the lower, higher and highest group of the standardized mortality ratio was significantly higher than that in the lowest group (*P* < 0.05) (Fig. 1e). 6-h C_ssc_ in the higher group was significantly higher than that in the lower group (*P* < 0.05) (Fig. 1e). Completion of each sub-index of hour-3 bundle in the lower, higher and highest group was significantly higher than that in the lowest group (*P* < 0.05) (Fig. 1e). Completion of repeated measurement of lactate levels in patients with initial hyperlactatemia in the lower, higher and highest group was significantly higher than that in the lowest group (*P* < 0.05) (Fig. 1e). Completion of resuscitation with vasopressor in patients with MAP ≤ 65 mmHg after fluid resuscitation and completion of CVP and ScvO2 were measured in patients with lactate ≥ 4 mmol/L in the higher and highest group were significantly higher than that in the lowest group (*P* < 0.05) (Fig. 1e). These results indicated that lower standardized mortality ratio mean better *C*_ssc_.

## Discussion

On March 15, 2012, the Ministry of Health of China approved that Peking Union Medical College Hospital establish China-NCCQC. The Quality Improvement of Critical Care Program, led by China-NCCQC, was initiated in 2015. This study is part of the above program. *C*_ssc_ is closely related to patient prognosis [6]. At present, researches on *C*_ssc_ mainly focus on the relationship between *C*_ssc_ and prognosis [8, 9], and there are few researches on *C*_ssc_ itself and its influencing factors [10, 11]. How to improve *C*_ssc_ becomes an important part of clinical practice. Wang found that the *C*_ssc_ of emergency physicians is often hindered by the doctors' awareness and attitudes [12]. ICU structure indicators could affect the incidence of ICU-acquired infections and clinical outcomes [13]. Therefore, we designed this study to investigate the relationship between structure indicators of ICU and *C*_ssc_ in China. In our previous study, a multifaceted Q_ICU_ intervention was effective in improving 3 and 6-h *C*_ssc_ in septic shock in China [14]. In this study, we found that 6-h *C*_ssc_ was lower than 3-h *C*_ssc_. In the subgroup, completion of empiric broad-spectrum therapy was the highest, and completion of CVP and ScvO2 measured in patients with lactate ≥ 4 mmol/L was the lowest, which might be why 6-h *C*_ssc_ is lower than 3-h *C*_ssc._ The key to improving 6-h *C*_ssc_ and even the whole *C*_ssc_ is improving completion of CVP and ScvO2 measured in patients with lactate ≥ 4 mmol/L. In the 3-h subgroup, completion of appropriate routine microbiologic cultures (including blood) before starting antimicrobial therapy was the lowest. The key to improve 3-h *C*_ssc_ is improving completion of appropriate routine microbiologic cultures (including blood) before starting antimicrobial therapy.

Specifically, 3 and 6-h *C*_ssc_ is related to predicted mortality rate, standardized mortality ratio. 3 and 6-h *C*_ssc_ was better when the predicted mortality rate was higher, and the standardized mortality ratio was lower. The higher that the predicted mortality rate is, the higher that the proportion of patients admitted to the ICU with severe diseases is. The lower that the standardized mortality ratio is, the higher that the diagnosis and treatment level in the ICU is [15–17]. The combination of the above two indicators can fully reflect the medical level of an ICU. Higher levels lead to better *C*_ssc_.

The relationship of 3 and 6-h *C*_ssc_ with the proportion of ICU patients among total inpatient bed occupancy, the proportion of APACHE II score ≥ 15 in all ICU patients is uncertain and further research is needed. Interestingly, predicted mortality rate, which is closely related to APACHE II score is related to 3 and 6-h *C*_ssc_. This phenomenon might reflect the difference in test titers between the two indicators. When examining 3 and 6-h *C*_ssc_, predicted mortality rate might be a more effective indicator. 

While the relationship of 3 and 6-h *C*_ssc_ with the proportion of ICU patients among total inpatient bed occupancy is uncertain, there was no relationship of 3 and 6-h *C*_ssc_ with the proportion of ICU patients among total inpatients. Reason for the above phenomenon may be that both the proportion of ICU patients among total inpatient bed occupancy and *C*_ssc_ are correlated with hospital treatment level, while the proportion of ICU patients among total inpatients is not correlated with hospital treatment level.

The factors confounding the study include the equipment in ICU, the transport capacity of the hospital, the cooperation of relevant departments, the technical level of medical staff and so on. For example, whether there is lactate detection equipment in the ICU, whether the field staff can send samples to the relevant testing departments in time, and whether the relevant testing departments can conduct tests immediately after receiving the samples can significantly affect the monitoring of lactate. It can be seen from our investigation that the completion of lactate concentration measurement was lower than the completion of empiric broad-spectrum therapy. The latter can be done independently in the ICU. The completion of CVP and ScvO2 measurement, which required the highest technical level of medical staff, was the lowest among the items of 3 and 6-h *C*_ssc_.

There are some limitations of our study. First, since only 1 year of data was included in this study, the relationships of the structure indicators of ICU on 3 and 6-h *C*_ssc_ could not be analyzed continuously and dynamically. Second, those hospitals enrolled from China-NCCQC might be more motivated to improve sepsis care quality than other hospitals. Further studies will be necessary to determine the relationships of the structure indicators of ICU on 3 and 6-h *C*_ssc_ in hospitals in China that differ in characteristics from those that participated.

## Conclusions

The key to improving 6-h *C*_ssc_ and even the whole *C*_ssc_ is to improve completion of CVP and ScvO2 being measured in patients with lactate ≥ 4 mmol/L. The key to improve 3-h *C*_ssc_ is to improve completion of appropriate routine microbiologic cultures (including blood) before starting antimicrobial therapy. The factors influencing 3 and 6-h *C*_ssc_ in China are the predicted mortality rate and the standardized mortality ratio.

## Supplementary Information

**Additional file 1**: Basic information of hospitals in different provinces and cities. a = Hospitals, b = Beds, c = ICU beds, d = Patients admitted in hospitals, e = Patients admitted in ICUs, f = Days of hospital bed occupancy by patients, g = Days of ICU bed occupancy by patients, h = ICU doctor number, i = ICU nurse number.

**Additional file 2**: **Figure S1** 3 hours SSC bundles compliance rate (%) of hospitals in different provinces and cities. **Figure S2** Compliance rate of lactate concentration was determined in different provinces and cities. **Figure S3**. Compliance rate of microbiologic cultures before antimicrobial therapy in different provinces and cities. **Figure S4**. Compliance rate of empiric broad-spectrum therapy in different provinces and cities.** Figure S5.** Compliance rate of resuscitation with 30 ml/kg crystal liquid in different provinces and cities.

**Additional file 3**: **Figure 1**. 6 hours SSC bundles compliance rate (%) of hospitals in different provinces and cities. **Figure 2**. Compliance rate of retest of lactate levels in patients with initial hyperlactatemia in different provinces and cities. **Figure 3**. Compliance rate of resuscitation with vasopressor if MAP ≤ 65mmHg after fluid resuscitation in different provinces and cities. **Figure 4**. Compliance rate of CVP and ScvO2 were measured in patients with lactate ≥ 4mmol/L in different provinces and cities.

## Data Availability

The datasets supporting the conclusions of this article are included within the article and its additional files.

## Abbreviations

SSC: Surviving sepsis campaign
*C*_ssc_: Compliance with the surviving sepsis campaign guidelines
ICU: Intensive care unit
China-NCCQC: China National Critical Care Quality Control Center
MAP: Mean arterial pressure
CVP: Central venous pressure
ScvO2: Central venous oxygen saturation
