# Supplementary material for: Analysis of structure indicators influencing 3-h and 6-h compliance with the surviving sepsis campaign guidelines in China: a systematic review
Source: Eur J Med Res. 2021 Mar 19;26:27. doi: 10.1186/s40001-021-00498-7 (PMC7976719; doi:10.1186/s40001-021-00498-7)
Supplement: Supplementary file 1 — Additional file 1: Basic information of hospitals in different provinces and cities. a = Hospitals, b = Beds, c = ICU beds, d = Patients admitted in hospitals, e = Patients admitted in ICUs, f = Days of hospital bed occupancy by patients, g = Days of ICU bed occupancy by patients, h = ICU doctor number, i = ICU nurse number. [file 40001_2021_498_MOESM1_ESM.docx]

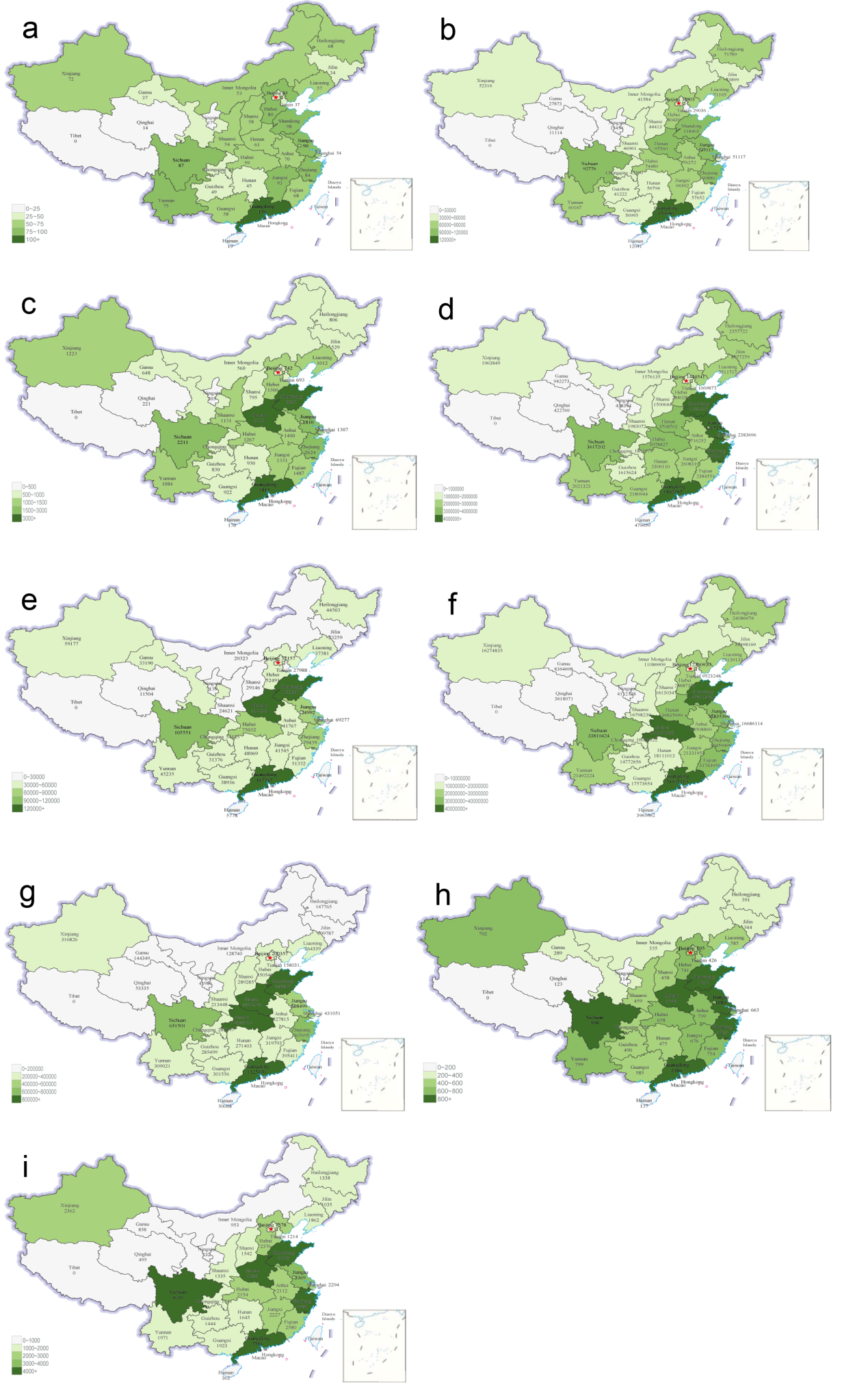


**Basic information of hospitals in different provinces and cities**. a = Hospitals, b = Beds, c = ICU beds, d = Patients admitted in hospitals, e = Patients admitted in ICUs, f = Days of hospital bed occupancy by patients, g = Days of ICU bed occupancy by patients, h = ICU doctor number, i = ICU nurse number.
