# Supplementary material for: Analysis of structure indicators influencing 3-h and 6-h compliance with the surviving sepsis campaign guidelines in China: a systematic review
Source: Eur J Med Res. 2021 Mar 19;26:27. doi: 10.1186/s40001-021-00498-7 (PMC7976719; doi:10.1186/s40001-021-00498-7)
Supplement: Supplementary file 2 — Additional file 2: Figure S1 3 hours SSC bundles compliance rate (%) of hospitals in different provinces and cities. Figure S2 Compliance rate of lactate concentration was determined in different provinces and cities. Figure S3. Compliance rate of microbiologic cultures before antimicrobial therapy in different provinces and cities. Figure S4. Compliance rate of empiric broad-spectrum therapy in different provinces and cities. Figure S5. Compliance rate of resuscitation with 30 ml/kg crystal liquid in different provinces and cities. [file 40001_2021_498_MOESM2_ESM.docx]

Figure 1. 3 hours SSC bundles compliance rate (%) of hospitals in different provinces and cities.

Figure 2. Compliance rate of lactate concentration was determined in different provinces and cities.

Figure 3. Compliance rate of microbiologic cultures before antimicrobial therapy in different provinces and cities.

Figure 4. Compliance rate of empiric broad-spectrum therapy in different provinces and cities.

Figure 5. Compliance rate of resuscitation with 30ml/kg crystal liquid in different provinces and cities.
