# Supplementary material for: Analysis of structure indicators influencing 3-h and 6-h compliance with the surviving sepsis campaign guidelines in China: a systematic review
Source: Eur J Med Res. 2021 Mar 19;26:27. doi: 10.1186/s40001-021-00498-7 (PMC7976719; doi:10.1186/s40001-021-00498-7)
Supplement: Supplementary file 3 — Additional file 3: Figure 1. 6 hours SSC bundles compliance rate (%) of hospitals in different provinces and cities. Figure 2. Compliance rate of retest of lactate levels in patients with initial hyperlactatemia in different provinces and cities. Figure 3. Compliance rate of resuscitation with vasopressor if MAP ≤ 65mmHg after fluid resuscitation in different provinces and cities. Figure 4. Compliance rate of CVP and ScvO2 were measured in patients with lactate ≥ 4mmol/L in different provinces and cities. [file 40001_2021_498_MOESM3_ESM.docx]

Figure 1. 6 hours SSC bundles compliance rate (%) of hospitals in different provinces and cities.

Figure 2. Compliance rate of retest of lactate levels in patients with initial hyperlactatemia in different provinces and cities.

Figure 3. Compliance rate of resuscitation with vasopressor if MAP ≤ 65mmHg after fluid resuscitation in different provinces and cities.

Figure 4. compliance rate of CVP and ScvO2 were measured in patients with lactate ≥ 4mmol/L in different provinces and cities.
